# Placenta Accreta Spectrum Leading to Peripartum Hysterectomy: A Case Report

**DOI:** 10.7759/cureus.83253

**Published:** 2025-04-30

**Authors:** Silky Dahiwale, Savita Somalwar, Anuja Bhalerao

**Affiliations:** 1 Department of Obstetrics and Gynaecology, NKP Salve Institute of Medical Sciences and Research Centre, Nagpur, IND

**Keywords:** peripartum hysterectomy, placenta accreta spectrum, placental anomalies, postpartum haemorrhage, retained placenta

## Abstract

Placenta accreta spectrum (PAS) is a significant obstetric complication characterized by abnormal invasion of the placenta into the uterine wall. It often necessitates a peripartum hysterectomy to prevent life-threatening hemorrhage. This case report highlights a rare presentation of PAS and the subsequent surgical intervention. A 31-year-old (gravida 4, para 1) woman at 26 weeks of gestation presented with abdominal pain. Ultrasonography and MRI revealed signs consistent with retained placenta following an intrauterine fetal death. Despite attempts at manual placental removal, the placenta was adherent, and an emergency hysterectomy was performed due to placenta accreta. The patient had a history of previous cesarean section, which likely contributed to the development of PAS. Intraoperatively, the distorted uterine anatomy further complicated the surgical approach. Hence, this case underscores the importance of early detection of PAS and appropriate surgical intervention to manage the associated risks. Comprehensive preoperative planning and a multidisciplinary approach are crucial to improving outcomes in cases of PAS.

## Introduction

Placenta accreta spectrum (PAS) is an abnormal invasion of trophoblast into the myometrium of the uterine wall that includes placenta accreta, placenta percreta, and placenta increta [1]. Placenta accreta accounts for 63% of cases, placenta percreta for 22%, and placenta increta for 15% [2]. Since the placenta cannot detach spontaneously, it can lead to continuous bleeding, making the condition life-threatening and often requiring a peripartum hysterectomy.

To prevent mortality, peripartum hysterectomy is frequently performed. To enhance obstetric care and, therefore, maternal health, further understanding of the causes, treatments, and adverse outcomes of peripartum hysterectomies is essential. The removal of the uterus within a certain amount of time after the delivery of the baby is known as a peripartum hysterectomy [3-5] and is mainly performed for postpartum hemorrhage (PPH). The rates of peripartum hysterectomies differ widely across the world [6]. In high-income nations, these rates are usually low, ranging from 0.26/1000 births in Denmark to 1.07/1000 deliveries in Italy [7]. As a result, several studies have shown an increase in peripartum hysterectomies [3,8]. The most prevalent reason for peripartum hysterectomy is placental anomalies in the current scenario [9]. Hence, the present report highlights a case of peripartum hysterectomy following placenta accreta.

## Case presentation

Patient information

A 31-year-old female, married for eight years, presented to the department of obstetrics and gynecology with the chief complaint of abdominal pain for one day. The menstrual history reported by the patient stated that the last day of the menstrual period was 31st December 2022, and the expected delivery date was 7th October 2023. The patient reported that the previous cycles were regular with a duration of bleeding consisting of three to four days, and a cycle length of 28-30 days, with an average flow. The obstetric history reported by the patient stated G4P1L1E1A1, which narrates that the patient had four pregnancies, resulting in one live birth through cesarean section that reached viability, one ruptured ectopic pregnancy with right-sided salpingectomy, and one abortion/miscarriage. Additionally, the current gestational age of the fetus was 26 weeks and three days, and the patient perceived the fetal movements well.

Clinical examination

The patient was vitally stable. The per abdominal examination revealed a uterus of size 24-26 weeks, with the presence of external ballottement, normal fetal heart sounds, scar of previous cesarean section, and no scar tenderness. Additionally, per speculum examination reported a healthy cervix vagina, and per vaginum assessment revealed closed os. The laboratory results were found to be within normal limits. But on the night of hospital admission, the patient presented with contractions and entered spontaneous labor.

Diagnostic assessment

For diagnostic purposes, ultrasonography (USG) was performed at the time of admission, which reported a single live intrauterine pregnancy of 25 + 2 weeks, cephalic in presentation, and with adequate liquor. Additionally, the estimated fetal weight was 780 grams, which was within the expected range for 25 + 2 weeks of gestation.

Therapeutic intervention

As the patient entered spontaneous labor, under the active assistance of personnel (AAP), the patient was positioned in the lithotomy position, and the baby was delivered in the vertex position through spontaneous vaginal delivery. However, the baby did not cry and was handed over to a pediatrician. Additionally, the placenta and membranes were not delivered spontaneously, and the placenta was retained. Unfortunately, the female baby was stillborn, as indicated by the intrauterine death of the baby. The baby weighed 780 grams, suggesting extreme prematurity or growth restriction.

Under AAP, general anesthesia (GA), and lithotomy position, Betadine painting and draping were done. Manual removal of the placenta was tried, but it was found to be adherent, possibly reporting a condition of PAS, where it was abnormally attached to the uterine wall, making manual removal difficult and dangerous; therefore, the procedure was abandoned and the patient was taken for USG of the pelvis along with Doppler. The USG report for the pelvis revealed a hyperechoic placenta-like structure of size 5.9 x 7.6 x 11.2 cm in the uterine cavity, which showed minimal vascularity. The uterus measured 7.5 x 9.4 x 18.7 cm and appeared bulky and enlarged in size, shape, and echopattern, suggestive of postpartum condition, but could also suggest other conditions like retained placental fragments. Additionally, no evidence of free fluid in the abdomen and pelvis was observed. Moreover, a minimally distended urinary bladder with a Foley bulb in situ was observed. The pouch of Douglas and the adnexa were found to be clear.

**Figure 1:**
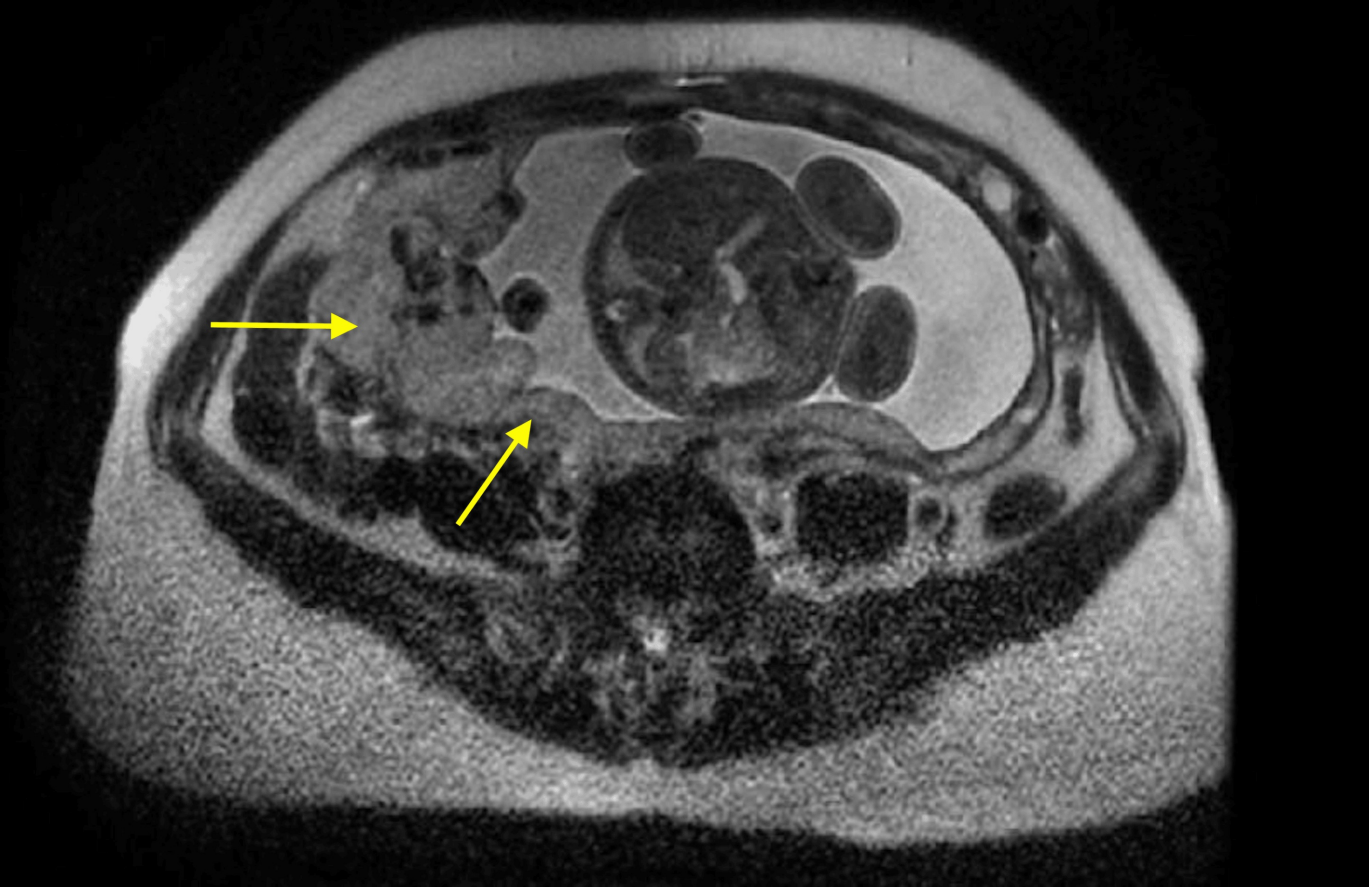
Axial T2-weighted image with arrows pointing at heterogeneously hyperintense fundo-posterior myometrium of the uterus.

Furthermore, magnetic resonance imaging (MRI), both plain and contrast of the pelvis, was performed, which showed that the uterus measured 18 x 9 x 6.5 cm (cranio-caudal x transverse × antero-posterior) in dimension and appeared bulky and heterogenous in signal intensity. A heterogeneous altered signal intensity area of size 9.4 x 6.1 x 5.7 cm (cranio-caudal, antero-posterior, and transverse dimensions, respectively) was noted in the fundo-posterior myometrium of the uterus that appeared predominantly iso-intense to hyperintense on T1-weighted images (T1WI) and heterogeneously hyperintense on short TI inversion recovery (STIR) and T2-weighted images (T2WI) for which axial view of T2WI is illustrated in Figure 1. Few of these areas appeared hypointense on T1WI and hyperintense on STIR and T2WI, suggestive of a cystic component. On diffusion-weighted imaging (DWI), some parts of these areas showed restriction that was suggestive of necrotic changes. The results were consistent with postnatal condition status, as these imaging features were suggestive of retained placenta. The patient and her husband were counseled regarding the need for an emergency obstetric hysterectomy, and after obtaining a written informed consent from the husband of the patient, the procedure was commenced.

The procedure was performed with AAP under GA and epidural anesthesia (EA). With the patient in a lithotomy position, an attempt to manually remove the placenta was made, but it could not be detached. The patient was repositioned in a supine position for abdominal surgery. Betadine painting and draping were done, and the abdomen and skin were opened by vertical incision. It was observed that the omentum had densely adhered to the anterior surface of the uterus. Hence, the adhesion was removed by sharp dissection. A subtotal hysterectomy was done in which the uterus with retained placenta was delivered completely, followed by vault closure and suspension. Part of the cervix was left behind as distorted anatomy could not be removed because the bladder was drawn up the right side. The patient received one pint of packed red cells (PRC) during the surgery, due to blood loss.

**Figure 2:**
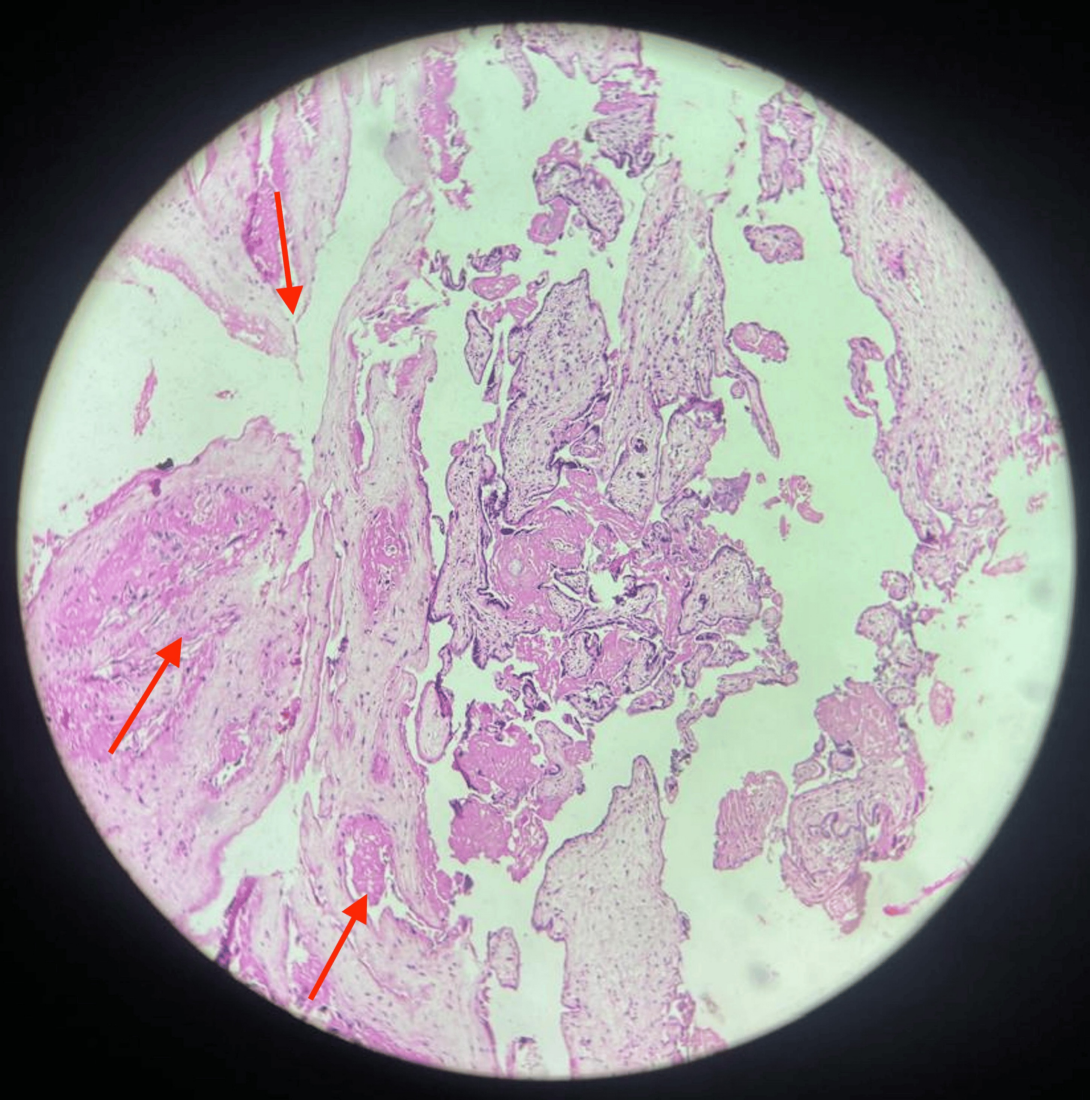
Histopathological examination with arrows illustrating chorioangiomatosis.

The intraoperative findings indicated several anatomical abnormalities, as a right corneal end, right-sided tubes, and ovaries could not be visualized. Additionally, the anatomy of the uterus was distorted, which was suggestive of the unicornuate uterus. However, the left-side tubes and ovaries were found to be normal. The bladder was drained up and was adherent to the lower uterine segment. The specimen, consisting of the uterus and the retained placenta, was directed for histopathological examination (HPE). The HPE results of the uterus with adherent placenta revealed placenta accreta, and the histological features of the placenta were suggestive of chorioangiomatosis, as shown in Figure 2.

## Discussion

PAS is an unusual invasion of the trophoblast into the myometrium of the uterine wall [1]. Patients with low-lying placenta and a previous history of cesarean section (CS) are considered to be at risk for the development of PAS. The most prevalent hypothesis of PAS is defective decidualization, which is defined as a thin, poorly formed, incomplete, nonexistent, or dysfunctional decidua. The endometrial-myometrial interface becomes scarred as a result of prior uterine surgery, which enables the placenta to attach to the myometrium directly or to invade it [10,11]. A multivariate analysis study revealed that the placenta previa was found to be an independent risk factor for PAS, whereas previous uterine surgery was not involved as a risk factor [1].

Additionally, the frequency of CS was found to be directly correlated with the frequency of PAS. Moreover, the incidence of PAS increased by 3% following primary CS [12]. Similarly, Palova et al. reported that the reason for the increasing rate of placenta accreta is attributed to the increasing rate of cesarean births, uterine curettage, and successful treatment of uterine atony with uterine drugs [13]. Furthermore, among the 122 women with placenta praevia in a retrospective cohort study, 25 had PAS, and 96% of them had previously undergone a CS [14].

The most common feature of PAS is bleeding when an attempt is made to separate the placenta. It may also be reported as antenatal bleeding as the placenta remains connected with the myometrium [1]. However, in the present case, the patient did not report a history of bleeding. The diagnosis of PAS during the antenatal period is essentially required for appropriate and adequate delivery of care. However, in the absence of risk factors, early detection of placental anomalies is challenging, and the diagnosis is often made only after the placenta is unsuccessfully removed after delivery [9].

The most frequently implemented intervention involves peripartum hysterectomy, which is used either to manage or prevent PPH. In a systematic review conducted by Jauniaux et al., peripartum hysterectomies were performed in 52.2% of cases, and 46.9% required blood transfusion among 7001 PAS cases [2]. Hence, in the present case, for the treatment of PAS, peripartum hysterectomy was performed, and, additionally, the patient required a blood transfusion, which corresponded well with the results of the previous studies.

## Conclusions

The present case highlights the complexity and severity of PAS, a life-threatening condition requiring prompt intervention to prevent maternal mortality, which necessitated an emergency peripartum hysterectomy following unsuccessful attempts at placental removal. This case underscores the importance of early antenatal diagnosis of PAS, particularly in women with known risk factors such as a history of cesarean deliveries. As saving lives is essential, these women need comprehensive, multidisciplinary, supportive care for the long term to enhance the treatment and the recovery process.
